# Changing Measles Epidemiology in Ghana, 2018–2023: Exploring Impact of COVID-19-Related Disruptions on Vaccination Coverage and Incidence

**DOI:** 10.3390/tropicalmed11100295

**Published:** 2026-10-16

**Authors:** Eunice Baiden-Laryea, Mercy Opare Addo, Joseph Asamoah Frimpong, Samuel Adolf Bosoka, Bernard Nkrumah, Rita Asante Kusi, Emmanuel Tettey Sally, Paul Henry Dsane-Aidoo, Abdul Gafaru Mohammed, Patrick Avevor, Horlali Gudjinu, Dennis Odai Laryea

**Affiliations:** 1 Department of Pharmacy and Pharmaceutical Sciences, College of Health Sciences, Kwame Nkrumah University of Science and Technology, Kumasi, Ghana; madupaddo@gmail.com; 2 African Field Epidemiology Network, Accra, Ghana; asamoah.frimpong@gmail.com (J.A.F.); bennkum@gmail.com (B.N.); angeasante@yahoo.com (R.A.K.); mohammedabdulgafaru46@gmail.com (A.G.M.); 3 Department of Epidemiology and Biostatistics, Fred. Binka School of Public Health, University of Health and Allied Science, Ho, Ghana; 4 Expanded Program on Immunization, Public Health Division, Ghana Health Service, Accra, Ghana; etsally1@gmail.com; 5 UNICEF Country Office, 4-8 Rangoon Close, Cantonment, Accra, Ghana; dsaneaidoo@gmail.com; 6 World Health Organization Ghana Country Office, 12 Agbaamo Street, Accra, Ghana; patavevor@yahoo.com; 7 Disease Surveillance Department, Public Health Division, Ghana Health Service, Accra, Ghana; sirhorlali@yahoo.co.uk (H.G.); denola@live.com (D.O.L.)

**Keywords:** measles, epidemiology, surveillance, vaccination, pandemics, Ghana

## Abstract

**Background**: Measles remains a public health threat in Ghana due to persistent immunity gaps and suboptimal vaccination coverage. The COVID-19 pandemic disrupted routine immunization and may have altered measles transmission. This study assessed measles burden, vaccination status of confirmed cases, and spatiotemporal trends in Ghana, and examined the pandemic’s impact on measles control. **Methods**: We analyzed measles cases reported in Ghana from 2018 to 2023, including age, sex, vaccination status, and region, alongside national measles–rubella (MR) vaccination coverage. Cumulative incidence was calculated as confirmed cases per million population. Interrupted time series analysis assessed pandemic-related disruptions in vaccination coverage and case reporting. Multivariable logistic regression identified factors associated with infection and vaccination status. **Results**: Ghana reported 1688 confirmed measles cases, a cumulative incidence of 51.9 per million, with a case fatality rate of 0.1%. Incidence rose from 1.1 per 1,000,000 in 2018 to 28.3 per 1,000,000 in 2023, with wide geographic variation. The odds of measles infection were higher among those aged 5–14 years (aOR = 1.41; 95% CI: 1.19–1.68, *p* < 0.001) and 15–44 years (aOR = 1.74; 95% CI: 1.37–2.22, *p* < 0.001) than among infants under one year. Compared with no vaccination, one dose was associated with lower odds of measles infection (aOR = 0.41; 95% CI: 0.34–0.48, *p* < 0.001), as was two doses (aOR = 0.38; 95% CI: 0.33–0.44, *p* < 0.001). MR1 coverage fell from 91.7% to 86.2%, and MR2 coverage stayed below the 95% herd immunity threshold throughout, with both showing an inverse relationship with incidence. The COVID-19 pandemic caused an immediate 64% decline in confirmed cases (IRR 0.36, 95%CI 0.17–0.75, *p* = 0.007). MR2 coverage dropped 3.66 percentage points at pandemic onset while MR1 rose slightly; neither change was significant. **Conclusions**: Measles burden in Ghana rose substantially between 2018 and 2023, with geographic variation and an inverse relationship between MR coverage and incidence. Suboptimal two-dose coverage and pandemic disruptions sustained transmission despite vaccination’s protective effect. Strengthening routine immunization and surveillance during emergencies is critical to detect outbreaks early and achieve measles elimination.

## 1. Introduction

Measles remains a major vaccine-preventable disease, with the greatest burden in low- and middle-income countries [1,2,3,4]. However, recent outbreaks in high-income countries show that it remains a global threat [5]. Despite the availability of an effective and affordable vaccine, measles causes periodic outbreaks due to gaps in routine immunization, population immunity, and surveillance [6]. In Ghana, measles vaccination is delivered primarily through the Expanded Programme on Immunization, with additional periodic supplemental immunization activities to achieve herd immunity [7,8]. However, measles transmission has persisted, indicating ongoing vulnerability within the population [9].

Measles control is a key indicator of routine immunization system performance and population immunity [10]. Its elimination is prioritised under Immunization Agenda 2030 and the Global Measles and Rubella Strategic Framework, which set a target of fewer than one confirmed case per million population per year [11]. Ghana’s measles incidence has previously been reported at 41.9 cases per million in 2019 [12], and recent surveillance data suggest a worsening trend, raising concerns about the country’s progress toward elimination. Persistent programmatic challenges including suboptimal two-dose coverage, cold-chain inefficiencies, and geographic inequities in service delivery continue to undermine population immunity and sustain transmission [9,13].

With the emergence of the COVID-19 pandemic in 2020, health systems were disrupted globally [14,15]. Public health resources were redirected toward pandemic response, while movement restrictions, fear of infection, and service interruptions affected access to routine health services. Immunization programmes were particularly affected, leading to missed vaccinations and delays in catch-up activities [16]. In Ghana, these disruptions were compounded by vaccine procurement and financing challenges in the post-pandemic period. Delays in government vaccine financing contributed to vaccine stockouts and declining immunization coverage in 2022–2023, with measles vaccine shortages reported in 2023 [17,18]. These disruptions may have widened immunity gaps and increased susceptibility to measles, raising concerns about the potential for outbreaks in Ghana.

Recent measles surveillance data in Ghana suggest temporal and geographic variation in disease occurrence before, during, and after the COVID-19 pandemic period [7,19]. Changes in incidence patterns across regions and age groups point to a shifting epidemiology that may be linked to pandemic-related disruptions in vaccination and surveillance [19,20]. Understanding how measles epidemiology has evolved over time, and how these changes relate to measles vaccination coverage during the COVID-19 period, is essential for guiding measles control and elimination efforts in Ghana.

Assessing the changing epidemiology of measles in the context of the COVID-19 pandemic is central to Ghana’s progress toward the Sustainable Development Goals, particularly SDG 3 on child survival and universal access to essential health services [10]. COVID-19-related disruptions to routine immunization threaten achievement of these targets by widening immunity gaps and increasing outbreak risk. Analysis of temporal and regional patterns in measles incidence supports targeted immunization strategies, outbreak preparedness, and efficient resource allocation while informing post-pandemic recovery and catch-up vaccination efforts recommended by the World Health Organization [21].

This study therefore examined the changing epidemiology of measles in Ghana from 2018 to 2023. Specifically, we aimed to: (1) describe the burden, demographic characteristics, and spatiotemporal distribution of confirmed measles cases; (2) identify factors associated with measles infection and vaccination status among confirmed cases; and (3) assess the impact of the COVID-19 pandemic on measles vaccination coverage and confirmed measles case counts using interrupted time series analysis.

## 2. Materials and Methods

### 2.1. Study Design

We conducted an analytical cross-sectional study by reviewing nationwide measles surveillance data collected at sub-national level between 2018 and 2023. Analytical approaches included multivariable logistic regression, and interrupted time series analysis. We conducted this analysis in April 2024.

### 2.2. Study Area

Ghana has an estimated population of over 34 million and is administratively divided into 16 regions and 261 districts, each comprising several sub-districts [22]. Measles surveillance is conducted through a tiered system spanning the community level (Community-based Health Planning and Services (CHPS), health centres and hospitals) to the district, regional, and national levels. The country is further classified into three ecological zones; northern, middle, and southern, within which regions share similar climatic and environmental characteristics that may influence disease transmission patterns. Ghana experiences two main seasons: a rainy season, which typically begins between April and May and ends between September and November depending on the region, and a dry season (locally known as the Harmattan), which starts between November and December and ends between March in some areas, extending to May or June in others. Although measles transmission can occur year-round, incidence generally peaks during the dry season.

### 2.3. Study Population and Selection Criteria

The study population was persons with clinically suspected and/or laboratory-confirmed measles reported between January 2018 to December 2023 from all 16 regions.

The study included records of all cases reported through the national surveillance system and excluded records of individuals with a misdiagnosis of measles or cases with significant missing or incomplete data.

### 2.4. Data Sources and Collection

We extracted data from the regional and national measles line list, case-based forms and the Surveillance Outbreak Response Management and Analysis System (SORMAS). SORMAS is an electronic public health surveillance and outbreak management platform. It is used to report and document investigation of and response to infectious disease outbreaks in real time in Ghana [23]. We also extracted aggregated data for each year from the District Health Information Management System II (DHIMS2) using the monthly Integrated Disease Surveillance Response (IDSR) reporting forms. Measles immunization data and projected population were also extracted from DHIMS2. The detailed data extraction procedure from DHIMS2 has been described previously in similar studies [24]. Variables of interest included age, sex, year, reporting region, laboratory results and outcome of case. Vaccination status was assessed using vaccination cards where available; caregiver recall was used when vaccination cards were unavailable. Data was accessed on 1 April 2024, and did not include any information that could identify individual participants during or after data collection.

### 2.5. Case Definition

The Ghana Integrated Disease Surveillance and Response Technical Guidelines (3rd Edition) assign the following case definitions for measles:

Suspected case: Any person with fever and maculopapular (non-vesicular) generalized rash and cough, coryza or conjunctivitis (red eyes) or any person in whom a clinician suspects measles.

Confirmed case: A suspected case with laboratory confirmation (positive IgM antibody or Polymerase Chain Reaction) or epidemiological link to confirmed cases in an outbreak [25].

### 2.6. Data Quality and Validity Checks

Data quality was assessed during data extraction and cleaning. We assessed the completeness of monthly IDSR reporting in DHIMS2 and case-based records in SORMAS. Reporting rates in DHIMS2 exceeded 95% throughout the study period. We also assessed the completeness of key study variables, including age, sex, district, date of symptom onset, vaccination status, and laboratory results. Extracted data were screened for duplicate records, missing values, invalid dates, and inconsistent values. Logical consistency between related variables was also assessed.

To assess consistency between surveillance systems, aggregated measles counts in DHIMS2 were compared with corresponding case-based records in SORMAS by district and reporting period. Identified discrepancies were reviewed and resolved using the available surveillance records. SORMAS also incorporates built-in data quality controls, including duplicate detection, standardized validation rules, and completeness prompts. Data were cleaned and standardized before analysis.

### 2.7. Data Management and Analysis

#### 2.7.1. Descriptive Analysis

We analysed all data using Stata/IC version 15 (StataCorp, College Station, TX, USA). We calculated the cumulative incidence rate as the total number of confirmed measles cases divided by the population at risk, expressed per one million population. Frequencies and proportions were used to describe measles cases by person-level characteristics. We performed spatiotemporal analysis to describe the distribution of cases by place and time. We used the Quantum Geographic Information System (QGIS), version 4.4.2 to map the spatiotemporal distribution of measles incidence and vaccination coverage.

#### 2.7.2. Factors Associated with Measles Positivity and Vaccination

We used binary logistic regression to examine factors associated with testing positive for measles and factors associated with receiving at least one dose of a measles-containing vaccine among confirmed cases (breakthrough cases). For the measles-positivity outcome, we applied a test-negative design restricted to suspected cases under surveillance, not general population controls. We recategorized laboratory results into two binary outcomes. Suspected cases with indeterminate or negative results were classified as non-measles. Suspected cases with IgM-positive results were classified as measles cases. For vaccination status, we defined vaccinated cases as those who had received at least one dose (dose 1 or dose 2) of measles-containing vaccine. We classified cases with unknown vaccination status and those who had not received any dose as unvaccinated. We reported adjusted odds ratios (aORs) with 95% confidence intervals. We defined statistical significance at *p* < 0.05.

#### 2.7.3. Interrupted Time Series Analysis

We used interrupted time series analysis (ITSA) to assess the effect of the COVID-19 pandemic on two measles-related outcomes: monthly confirmed measles case counts and monthly measles-rubella vaccination coverage (MR1 and MR2). The analysis covered January 2018 to December 2023. We defined March 2020 as the intervention point, marking the onset of the COVID-19 pandemic. The pre-pandemic baseline spanned January 2018 to February 2020. The pandemic period spanned March 2020 to December 2023.

#### 2.7.4. Impact of COVID-19 on Confirmed Measles Cases

We modelled monthly confirmed measles case counts using a negative binomial ITSA model with robust standard errors. We selected the negative binomial model over the Poisson model because the outcome data showed substantial overdispersion (α = 1.43). This violated the equidispersion assumption of the Poisson model. We adjusted the model for underlying secular trends and monthly seasonality using Fourier terms comprising pairs of sine and cosine functions. The final model demonstrated good overall fit (Wald χ^2^ = 95.35, *p* < 0.001). We reported results as incidence rate ratios (IRRs) with 95% confidence intervals, representing the multiplicative change in monthly confirmed measles case counts associated with each model parameter.

#### 2.7.5. Impact of COVID-19 on MR1 and MR2 Vaccination Coverage

We modelled MR1 and MR2 coverage separately using segmented regression. Each model included three core parameters. The time variable captured the underlying pre-pandemic trend. The intervention variable was a binary indicator estimating the immediate level change at pandemic onset. The time after variable estimated the change in monthly slope during the pandemic period, relative to the pre-pandemic trend.

Some MR1 and MR2 coverage values exceeded 100%. This likely reflected inaccuracies in target population denominators, migration, or inconsistent reporting practices. We modelled both outcomes on their original percentage scale to accommodate values above 100% and produces estimates interpreted as absolute percentage point changes.

#### 2.7.6. Model Diagnostics and Final Model Selection

We first fitted ordinary least squares regression models and examined the residuals using correlograms to assess the independence of errors. We detected significant autocorrelation at multiple lags in both MR1 and MR2 residuals, indicating that standard regression models were not appropriate for these time series data. We fitted both models within an autoregressive integrated moving average (ARIMA) framework using maximum likelihood estimation. We selected the final model structure for each outcome separately based on residual diagnostics, the Akaike Information Criterion (AIC), and the Bayesian Information Criterion (BIC).

For MR1, the ARIMA (1, 0, 0) model representing a first-order autoregressive process produced the lowest AIC (533.6) and BIC (547.3). Adding a seasonal autoregressive term at lag 12, referred to as SARIMA (1, 0, 0, 12), did not improve model fit for MR1, as the seasonal AR term was not statistically significant (*p* = 0.241) and both AIC (533.7) and BIC (549.6) increased. We therefore selected ARIMA (1, 0, 0) as the final model for MR1.

For MR2, the ARIMA (1, 0, 0) model with a seasonal autoregressive term at lag 12, referred to as SARIMA (1, 0, 0, 12), produced lower AIC (538.9) and BIC (554.9) than the model without the seasonal term, and the seasonal AR term was statistically significant (*p* = 0.036). This indicated that MR2 coverage exhibited meaningful annual cyclical patterns that required explicit modelling. We therefore selected ARIMA (1, 0, 0) with SARIMA (1, 0, 0, 12) as the final model for MR2. Adding a second autoregressive term did not improve fit in either outcome or was excluded from both final models.

We validated both final models by re-examining the residuals using correlograms. All Ljung–Box Q statistic *p*-values across 34 lags exceeded 0.05 for both models, confirming that autocorrelation and seasonal patterns had been fully accounted for and that both models were adequately specified. We acknowledge that the pre-pandemic baseline of 26 months, while meeting the minimum threshold for ITSA, may limit the precision of pre-pandemic trend estimates, and interpreted findings accordingly.

## 3. Results

### 3.1. Demographic Characteristics of Reported Measles Cases

**Figure 1:**
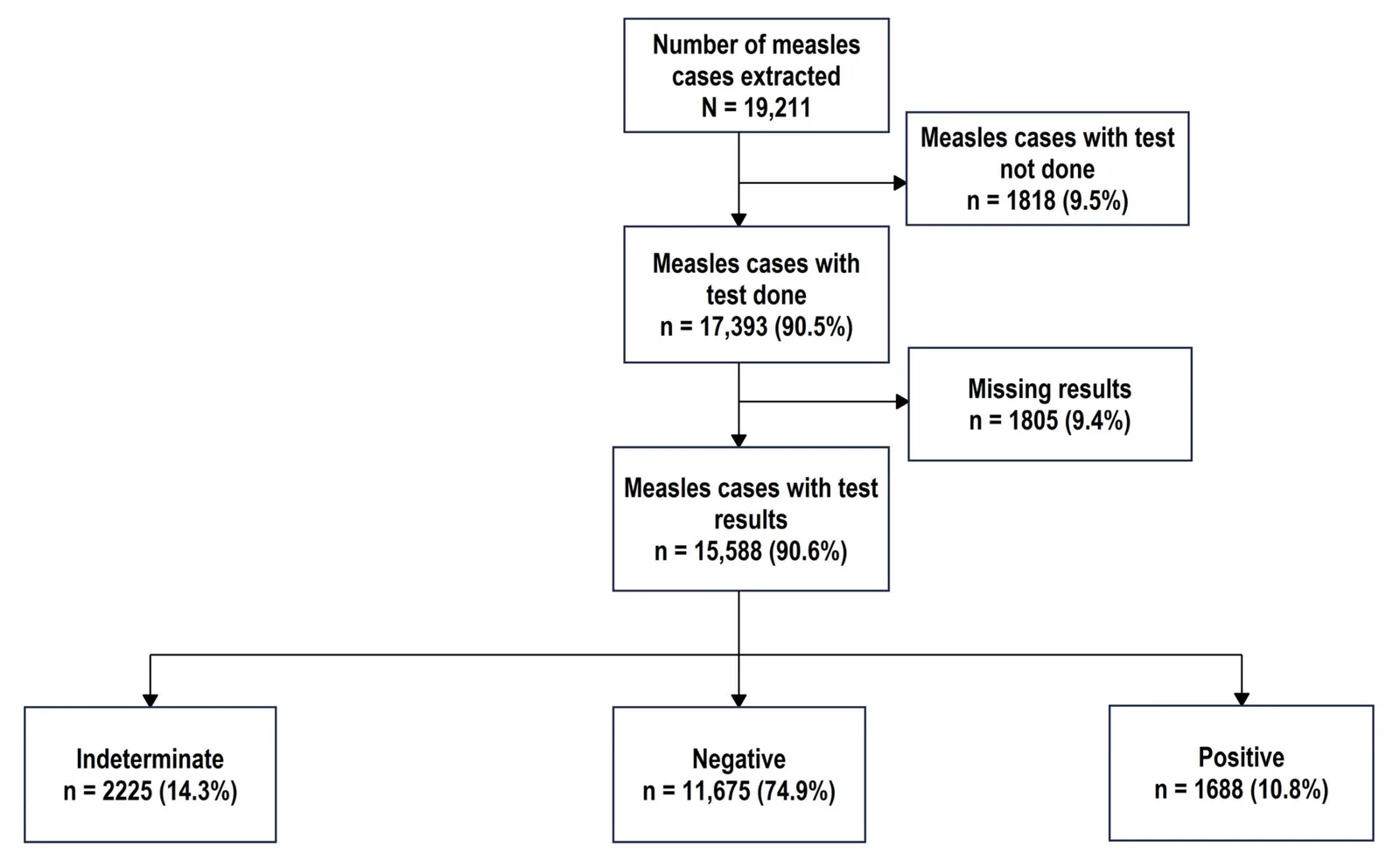
Measles case extraction and laboratory classification, Ghana, 2018–2023.

Of the 19,211 suspected measles cases identified, 90.5% (17,393) underwent laboratory testing. Among those tested, 89.6% (15,588) had complete serological results, while 10.4% (1805) had missing results. Of cases with complete serological data, 14.3% (2225/15,588) had indeterminate results and 10.8% (1688/15,588) were IgM-positive for measles (Figure 1).

The median age of the confirmed cases was 2 (interquartile range (IQR) 0–7) years. Case-patients aged 1–4 years accounted 39.9% (673/1688) of the confirmed cases, while those aged 45 years and above accounted for 1.0% (14/1688). Males represented 52.5% (886/1688) of confirmed cases. By regional distribution, the Northern Region reported 27% (456/1688) of the confirmed cases, whereas the Oti Region reported 0.8% (13/1688) (Table 1).

### 3.2. Incidence and Mortality Rates of Measles in Ghana

**Figure 2:**
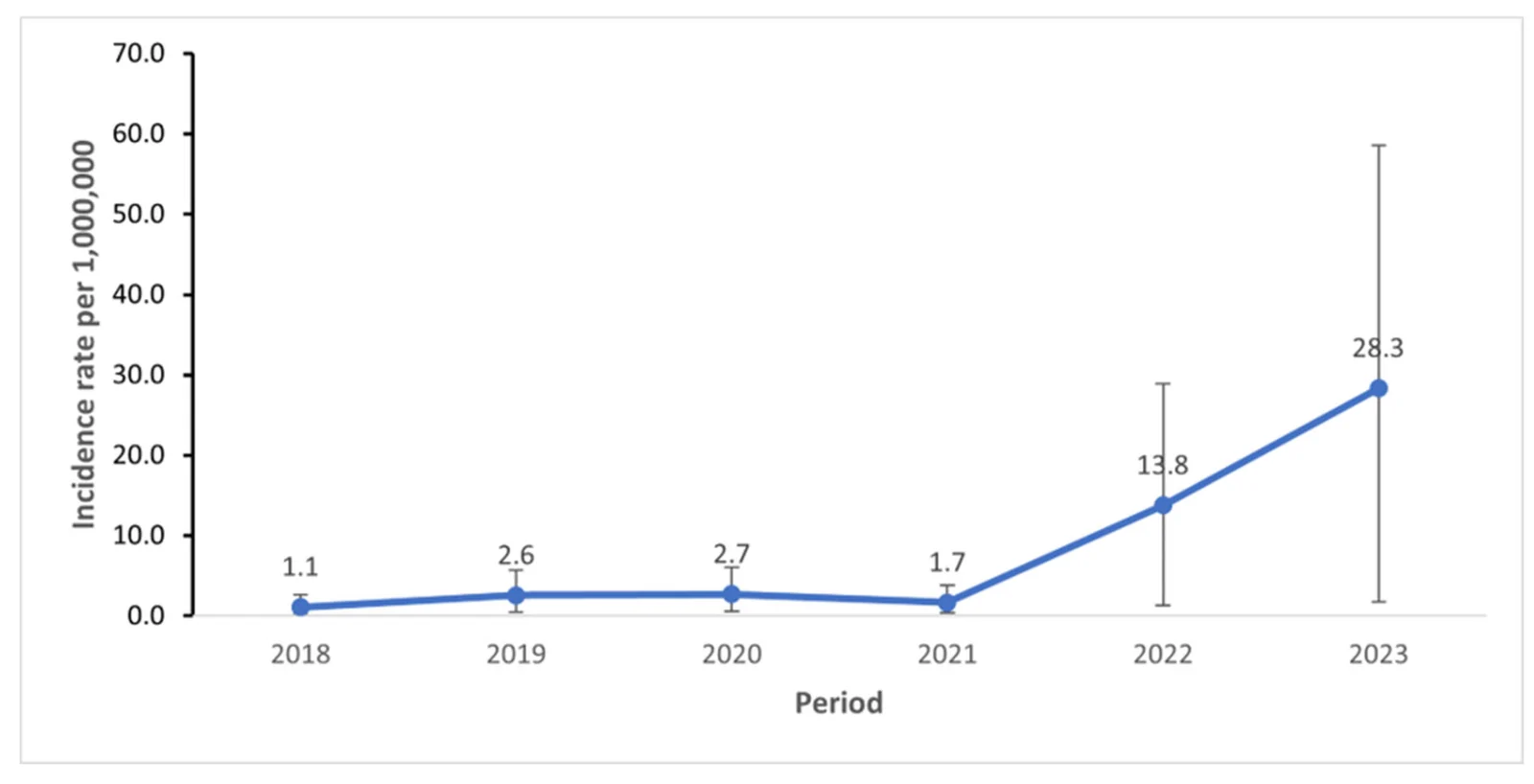
Annual incidence rate of measles, Ghana, 2018–2023. Error bars represent 95% Poisson confidence intervals.

The cumulative incidence rate of measles over the six-year period was 51.9 (95%CI: 49.40–54.40) cases per million. The incidence rate had increased from 1.1 (95%CI: 0.76–1.54) cases per million in 2018 to 28.3 (95%CI: 26.6–30.2) cases per million in 2023 (Figure 2). There were two (2) measles-related mortalities within the study period.

### 3.3. Spatiotemporal Distribution of Measles Incidence Rate in Ghana

**Figure 3:**
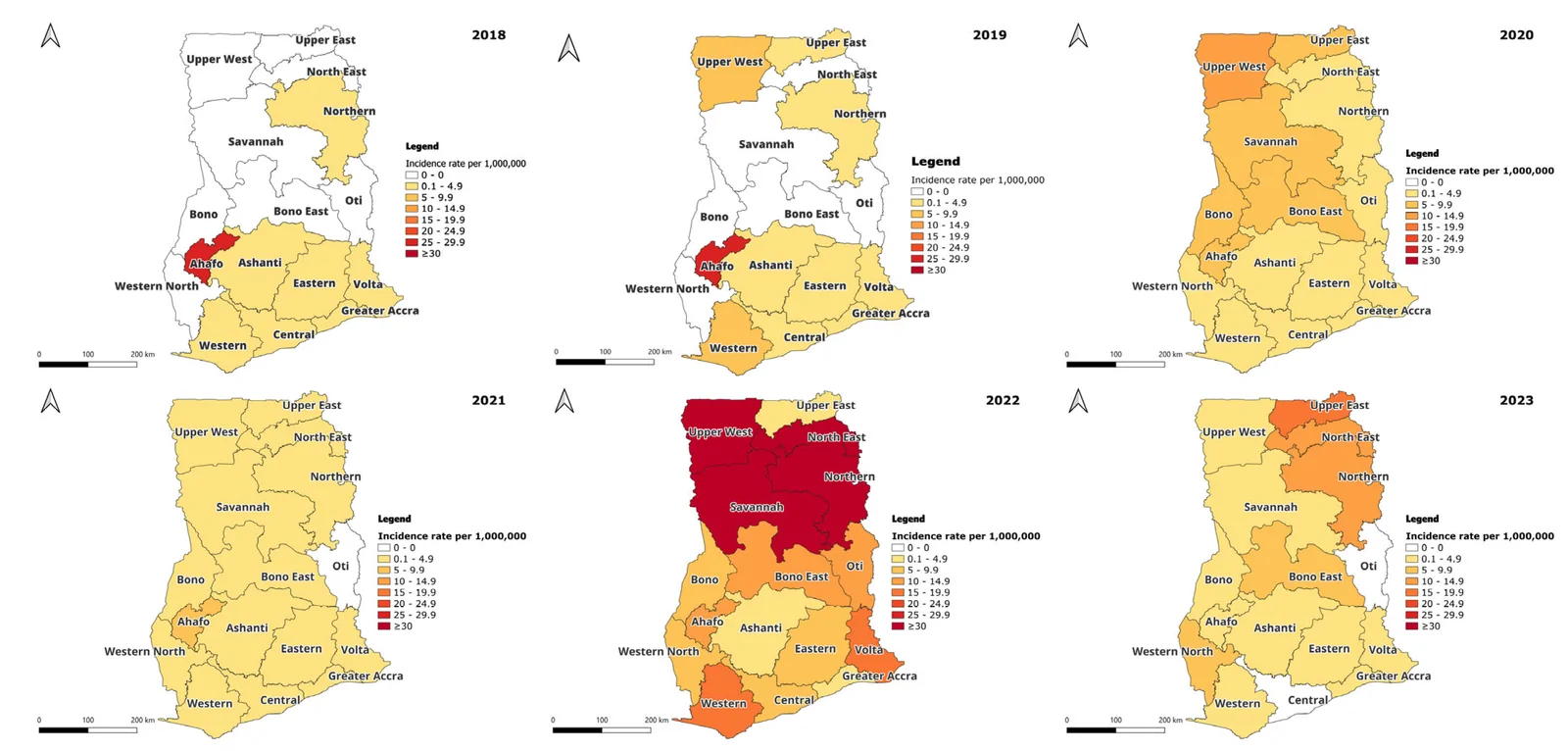
Spatiotemporal distribution of measles incidence rate in Ghana, 2018–2023.

The spatiotemporal analysis revealed that from 2018 to 2019, the incidence of measles was low across most regions except in Ahafo, which recorded the highest incidence rate of 26.1 and 27.4 cases per million in 2018 and 2019 years, respectively. However, in 2020 and 2021, measles incidence increased across several regions including Bono (8.8 per million), Bono East (5.3 per million), Upper West (13.8 per million), and Savannah (8.0 per million) regions. A surge in measles cases was observed in 2022, with the highest incidence recorded in Savannah (109.4 per million). A sharp decline was observed in most regions in 2023 with Upper East (17.9 per million) region recording the highest incidence followed by Northern (13.7 per million), and Northeast (11.0 per million) regions (Figure 3).

### 3.4. Seasonal Trend of Measles Cases

**Figure 4:**
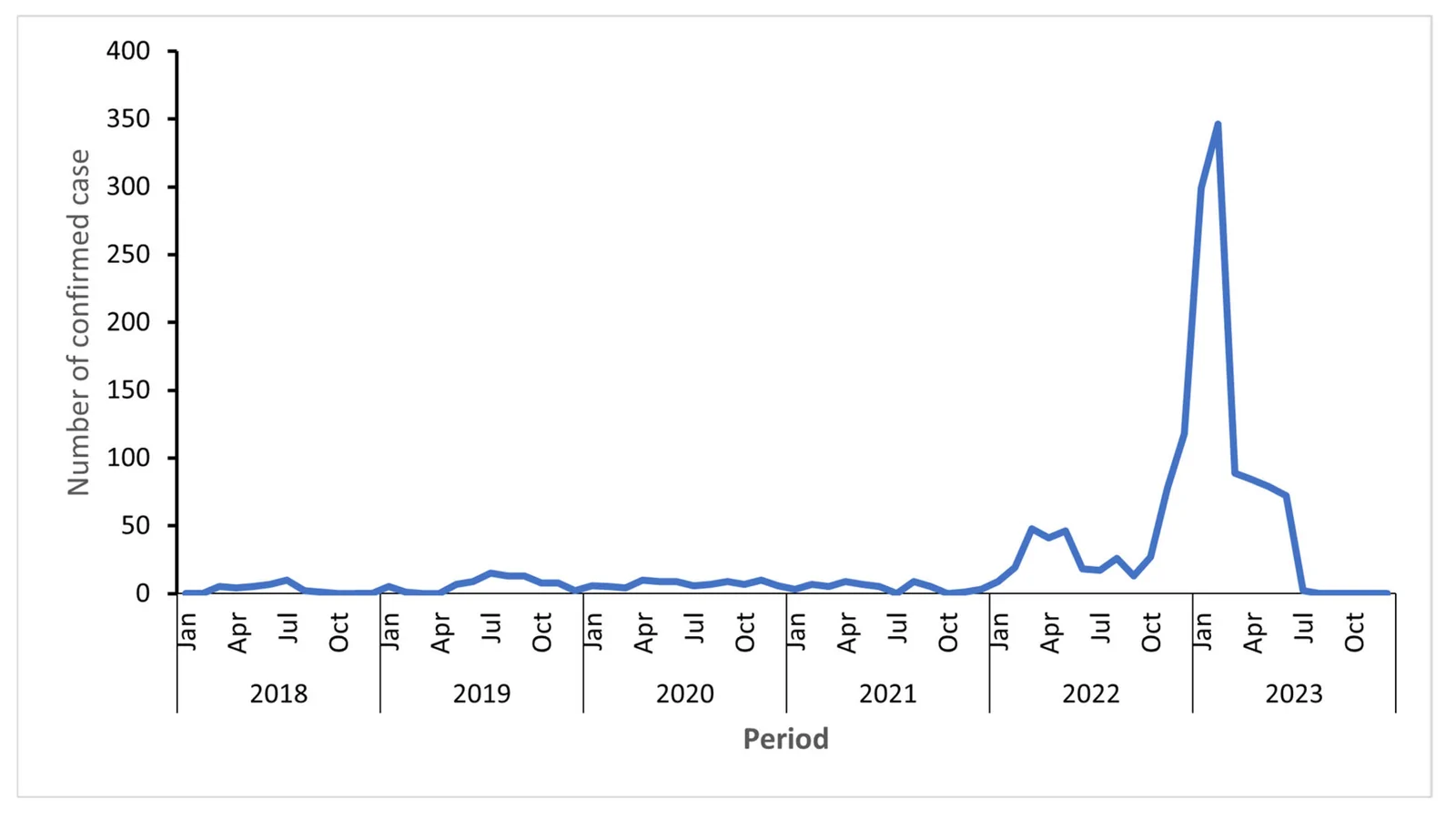
Distribution of confirm measles cases by year and month, Ghana, 2018–2023.

The trend of measles cases showed disease incidence all year round, with slight peaks in July 2018 and July 2019. Cases peaked again in March 2022, and within December 2022. The highest peak was observed in February 2023 (Figure 4).

### 3.5. Factors Associated with Measles Infection

Age was significantly associated with measles infection. Compared with children aged <1 year, individuals aged 5–14 years had 41% higher odds of measles infection (aOR = 1.41; 95% CI: 1.19–1.68, *p* < 0.001), while those aged 15–44 years had 74% higher odds (aOR = 1.74; 95%CI: 1.37–2.22, *p* < 0.001). In contrast, children aged 1–4 years had 6% lower odds of measles infection, although this difference was not statistically significant (aOR = 0.94; 95% CI: 0.81–1.10; *p* = 0.445). Similarly, individuals aged ≥45 years had 63% higher odds of measles infection, but the association was not statistically significant (aOR = 1.63; 95% CI: 0.93–2.85; *p* = 0.089). Meanwhile, sex was not significantly associated with being infected. Measles vaccination status was significantly associated with measles infection. Case-patients who received one dose (aOR = 0.41; 95%CI: 0.34–0.48, *p* < 0.001) and two doses of measles (aOR = 0.38; 95%CI: 0.33–0.44), *p* < 0.001) were 59% and 62% less likely to develop measles compared to case patients who were not vaccinated.

Compared with case-patients in the Ahafo Region, those residing in the Savannah Region had more than fourfold higher odds of measles infection (aOR = 4.41; 95%CI: 3.05–6.39, *p* < 0.001), those in the Northern and Northeast Regions had over fivefold higher odds (aOR = 5.10; 95%CI: 3.84–6.76), *p* < 0.001), and those in the Upper East Region had approximately 3.5-fold higher odds (aOR = 3.49; 95%CI: 2.58–4.73), *p* < 0.001), respectively. Meanwhile those residing in the Eastern Region had 42% lower odds of measles infection (aOR = 0.58; 95% CI: 0.40–0.84, *p* = 0.004), while those in the Greater Accra and Volta Regions each had approximately 45% lower odds (aOR = 0.55; 95% CI: 0.38–0.79, *p* = 0.001) and (aOR = 0.55; 95% CI: 0.38–0.81, *p* = 0.002), respectively (Table 2).

### 3.6. Measles Vaccination Coverage and Case Trends in Ghana

**Figure 5:**
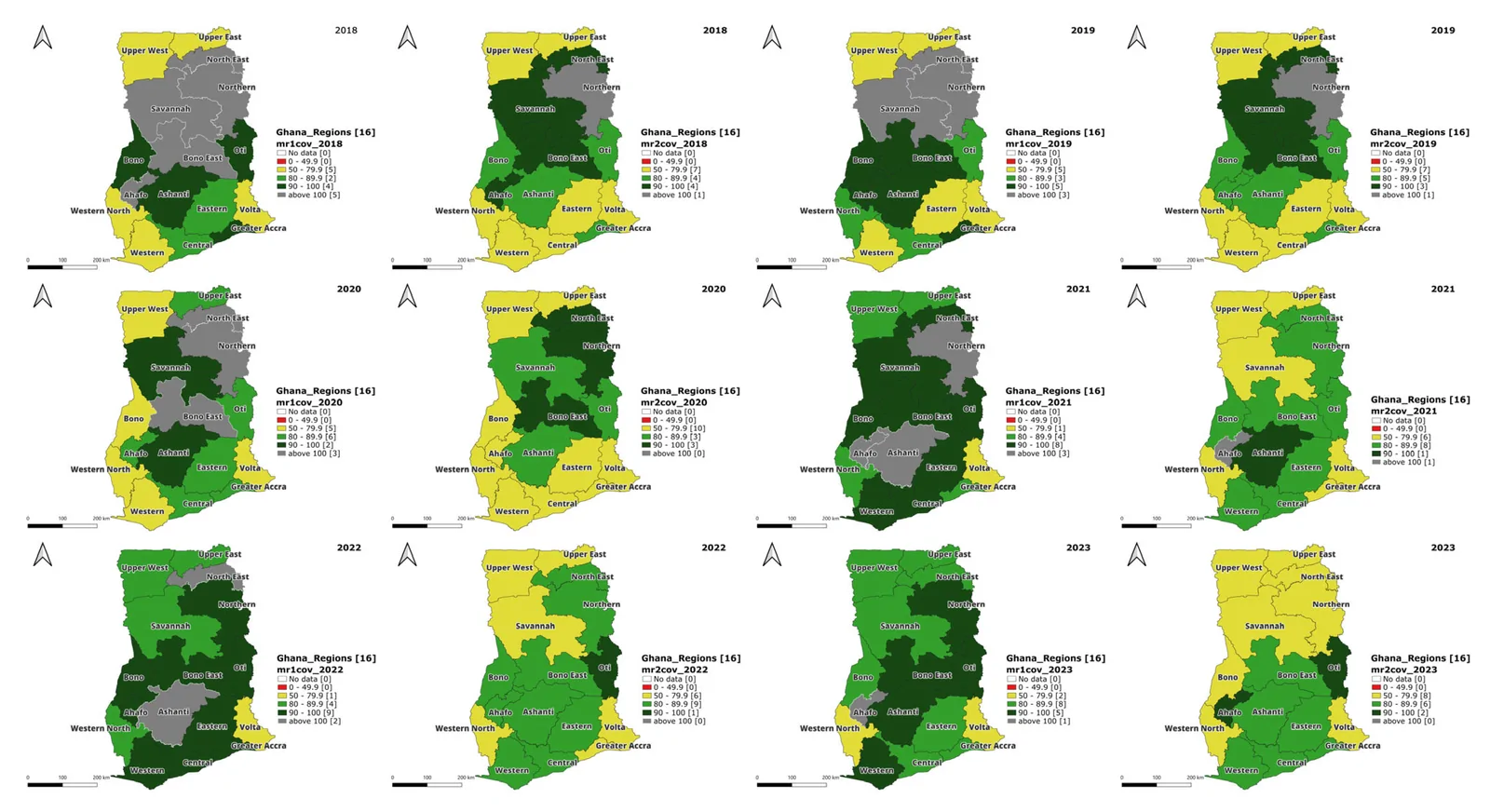
Measles vaccination coverage in Ghana, 2018–2023.

**Figure 6:**
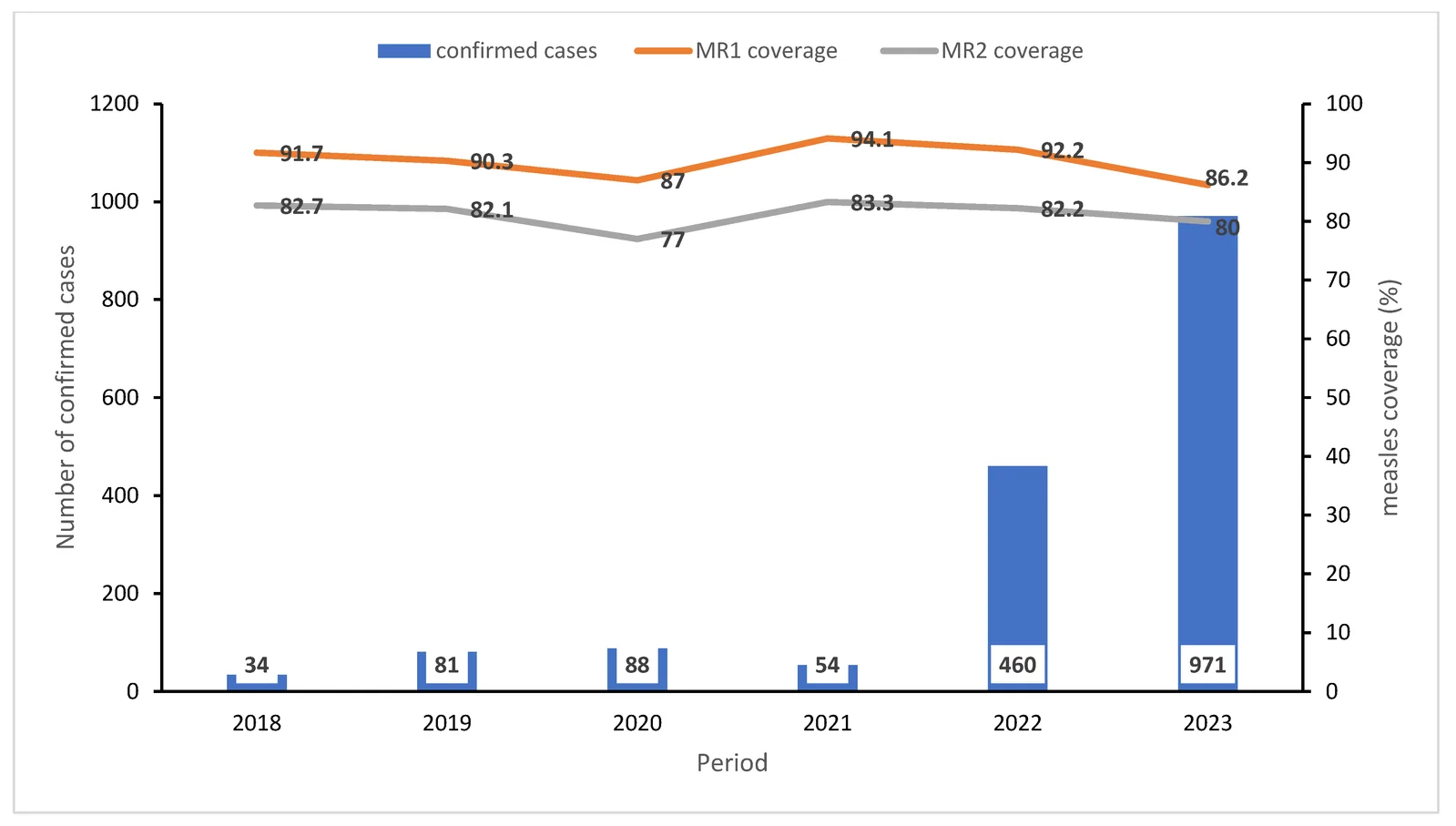
Trend of confirmed measles cases and MR1 and MR2 vaccination coverage, Ghana, 2018–2023.

Spatiotemporal analysis showed substantial regional variation in measles vaccination coverage. The Volta Region remained below the national 95% target for both doses throughout the study period, whereas the Northern Region consistently exceeded it (Figure 5). Nationally, MR1 coverage declined from 91.7% in 2018 to 86.2% in 2023, despite a temporary increase to 94.1% in 2021. MR2 coverage similarly declined from 82.7% to 80.0%, after peaking at 83.3% in 2021 (Figure 6). Confirmed measles cases showed an inverse temporal pattern. Cases increased from 34 in 2018 to 88 in 2020 as MR1 coverage declined to 87.0%, then decreased to 54 in 2021 as coverage increased to 94.1%. Cases subsequently rose sharply to 460 in 2022 and 971 in 2023 as MR1 coverage declined to 92.2% and 86.2%, respectively. MR2 coverage showed a similar pattern.

### 3.7. Vaccination Status and Its Predictors Among Confirmed Measles Cases

Vaccination was strongly protective against measles infection overall, yet breakthrough infections occurred among some vaccinated individuals. We examined vaccination history and its predictors among confirmed cases. Of 1688 confirmed cases, 39.5% (667/1688) had received at least one dose of measles-containing vaccine: 12.0% (203/1688) one dose and 27.5% (464/1688) two doses. Vaccination was highest among children aged 5–14 years (47.9%, 201/420) and 1–4 years (45.3%, 305/673). It was higher among males (40.2%, 356/886) than females (38.9%, 311/800). By region, coverage ranged from 58.9% (33/56) in the Eastern Region to 17.7% (17/96) in the Savannah Region.

Age and region of residence significantly predicted vaccination status. Children aged 1–4 years (aOR = 3.16; 95% CI: 2.37–4.22) and 5–14 years (aOR = 3.08; 95% CI: 2.25–4.23) were about three times more likely to be vaccinated than infants under one year. Children in the Upper West Region were more likely to be vaccinated than those in Ahafo (aOR = 2.08; 95% CI: 1.02–4.22). Children in Northeast, Northern, Savannah, and Upper East had 63%, 47%, 61%, and 58% lower odds of vaccination, respectively, compared with Ahafo (Table 3).

### 3.8. Impact of COVID-19 Pandemic on Measles Vaccination Coverage and Measles Cases

MR1 and MR2 vaccination coverage remained stable prior to the onset of the COVID-19 pandemic, with no significant pre-pandemic trends observed for MR1 (β_1_ = −0.21, 95% CI: −1.60 to 1.18) and MR2 (β_1_ = −0.10, 95% CI: −1.20 to 1.00). At the onset of the pandemic in March 2020, MR1 coverage increased slightly by 1.13 percentage points (95% CI: −19. to 21.51), while MR2 decreased by 3.66 percentage points (95% CI: −27.04 to 19.71), although neither change was statistically significant (Table 4). During the pandemic period, there was no evidence of a significant change in trend for MR1 (β_3_ = 0.24, 95% CI: −1.40 to 1.87) or MR2 (β_3_ = 0.26, 95% CI: −1.03 to 1.55). After adjusting for autocorrelation and seasonality using ARIMA models, MR2 exhibited a significant annual seasonal pattern, whereas MR1 did not (Table 4).

Before the COVID-19 pandemic, the monthly count of confirmed measles cases increased significantly by 6% (IRR = 1.06, 95% CI: 1.01 to 1.10, *p* = 0.015). At the onset of the COVID-19 pandemic in March 2020, confirmed measles cases declined immediately by 64% (IRR = 0.36, 95% CI: 0.17 to 0.75, *p* = 0.007). Following the pandemic onset, the monthly count of confirmed measles cases increased by 4% per month relative to the post-intervention trend (IRR = 1.04, 95% CI: 0.98 to 1.09, *p* = 0.152). This increase was not statistically significant. The monthly count of confirmed measles cases showed no significant seasonal variation, as none of the Fourier terms reached statistical significance (Table 5).

## 4. Discussion

This study assessed the burden and incidence of measles in Ghana by examining factors associated with measles positivity, vaccination status among confirmed cases, and national routine immunization coverages. We also compared measles incidence and vaccination patterns before and during the COVID-19 pandemic to quantify the extent of pandemic-related disruptions to measles control. Our study demonstrates how pandemic-related disruptions and existing population vulnerabilities sustained measles transmission and identifies key factors influencing measles outbreaks and vaccination uptake in Ghana.

Our study found a cumulative measles incidence of 51.9 cases per million population over the six-year period. This finding is comparable to the 60.3 cases per million reported for the WHO African Region from 2022 to 2023 [4], suggesting that the burden observed in our study is consistent with the high measles incidence reported across the region in recent years. Annual incidence in our study rose sharply, from 1.1 cases per million in 2018 to 28.3 cases per million in 2023. This increase points to intensifying measles transmission toward the end of the study period. The 2023 rate far exceeds the WHO elimination target of fewer than one confirmed case per million per year, and it signals ongoing active transmission [26].

This upward trend mirrors a previous multi-country analysis, which reported that measles incidence in Ghana rose from 0.8 cases per million in 2015 to 41.9 cases per million in 2019 [27]. Although the estimates are not directly comparable because the previous study used data from a multi-country analysis and covered a different period, the consistency in the upward trend suggests that measles incidence in Ghana has increased over time. In contrast, findings from South Africa showed a relatively low and fluctuating annual incidence between 2015 and 2020, ranging from 0.4 cases per million in 2015 and 2020 to a peak of 3.6 cases per million in 2017 [3]. Ghana’s rising incidence threatens progress toward both national and regional elimination goals [11]. Reversing this trend will require targeted supplementary immunization activities, stronger routine immunization, improved second-dose coverage, and enhanced surveillance and outbreak preparedness [27]. These measures are essential for advancing measles elimination in Ghana.

The spatiotemporal analysis revealed geographic variation in measles transmission across Ghana. Incidence remained low in most regions during 2018 and 2019. The Ahafo Region emerged as an early hotspot, likely reflecting localised immunity gaps or enhanced surveillance early detection. The rise in confirmed cases across multiple regions in 2020 and 2021 coincided with COVID-19-related disruptions to routine immunization and surveillance activities such as suspended supplementary immunization activities, reduced routine immunization visits due to movement restrictions or fear of infection [28], and reassignment of surveillance staff to COVID-19 response [29]. These disruptions likely delayed outbreak detection and created immunity gaps that facilitated measles spread across regions, a pattern also reported several studies in Africa [28]. The pronounced surge in 2022, particularly in the Savannah Region, likely reflects intensified transmission resulting from prolonged immunity gaps and missed catch-up vaccination opportunities. The persistent clustering of high incidence in northern regions highlights structural challenges in immunization service delivery and equitable access to health services in these areas.

These findings highlight the need for geographically targeted immunization strategies and strengthened subnational surveillance to rapidly identify and respond to emerging hotspots. Prioritising catch-up vaccination in high-burden and underserved regions is essential to close immunity gaps and prevent recurrent outbreaks.

Individuals aged 5 to 14 years and 15 to 44 years had significantly higher odds of measles infection compared with infants under one year. This pattern reflects substantial immunity gaps beyond early childhood, driven by waning immunity and incomplete uptake of the two-dose measles vaccination schedule, as documented in previous studies [1,30,31,32]. This pattern aligns with international evidence of suboptimal post-pandemic catch-up vaccination among young adults, indicating that persistent susceptibility in this age group is not unique to Ghana but reflects a broader post-COVID-19 challenge [33]. The introduction of the second measles dose in Ghana in 2012 means that cohorts of older children and young adults either missed the second dose altogether or received it later than recommended, leaving many inadequately protected. Operational challenges including cold-chain disruptions that reduce vaccine potency [34] and misclassification of vaccination status in routine surveillance records [35] may further contribute to the higher odds of measles observed among older age groups. Vaccination status ascertained through caregiver recall is particularly susceptible to inaccurate reporting, especially for older children or in settings with lower vaccination card retention, and this may partly explain the substantial regional variation in vaccination-related odds reported in this study.

Unlike age, sex was not a statistically significant predictor of measles infection, suggesting that observed case patterns are more likely influenced by caregiver health-seeking behaviour, vaccination uptake, and shared environmental exposures rather than inherent biological susceptibility. This finding supports sex-neutral immunization strategies [30,36,37,38].

Vaccination was strongly protective against measles, with vaccinated individuals showing 61% lower odds of infection. This finding is consistent with evidence from routine immunization programmes globally [6,30,39,40]. However, this individual-level protection has not translated into sufficient population immunity. National MR1 coverage declined from 91.7% in 2018 to 86.2% in 2023, while MR2 coverage remained persistently below the 95% herd immunity threshold required for elimination throughout the study period. The inverse relationship between declining MR coverage and increasing measles incidence clearly demonstrates that aggregate coverage figures mask important immunity gaps at the subnational level.

Short-term improvements in coverage in 2021 were followed by a temporary decline in cases, but measles resurged sharply in 2022 and 2023 as immunity gaps widened again. Persistent programmatic challenges and regional inequities, particularly in the northern regions, continue to slow progress toward Ghana’s 2030 measles elimination target [11]. These findings highlight the need for targeted region-specific strategies, catch-up vaccination campaigns, and stronger routine immunization systems to close immunity gaps and interrupt transmission.

Interrupted time series analysis showed no significant change in the level or trend of MR1 or MR2 coverage after the onset of COVID-19 in March 2020. Coverage remained stable, indicating a resilient immunization programme. However, the opposite direction of the immediate changes between MR1 and MR2 warrants attention. Although neither change reached statistical significance, the contrasting immediate responses of MR1 and MR2 at pandemic onset are noteworthy. MR1 coverage increased slightly, while MR2 declined by 3.66 percentage points. This directional difference suggests that second-dose vaccines may be more vulnerable to pandemic-related disruptions than first-dose vaccines. Caregivers may deprioritise second doses during periods of health facility avoidance or reduced service availability, particularly since MR2 is administered to older children who may be perceived as less at risk [9,41]. This pattern is consistent with evidence from other settings where second-dose coverage declined more sharply than first-dose coverage during health system disruptions [9,15,42,43,44]. Beyond physical access barriers, pandemic-related shifts in risk perception and generalized vaccine hesitancy likely contributed to reduced completion of routine booster schedules, a pattern documented in Italy [45]. The significant seasonal AR term in the MR2 model (beta = 0.38, *p* = 0.036) further confirms that second-dose coverage exhibits meaningful annual cyclical patterns, reflecting seasonal variation in health service utilisation and caregiver behaviour that is not present in first-dose coverage. However, these findings represent population-level patterns, and the ITS dataset did not include aggregated demographic characteristics to assess whether pandemic-related changes in vaccination coverage differed across demographic groups. The observed changes in measles epidemiology should also be considered in the context of disruptions to routine immunization during and after the COVID-19 pandemic. In Ghana, delayed vaccine procurement financing contributed to vaccine stockouts in 2022–2023, with measles vaccine shortages reported in 2023 [17]. These supply-side disruptions may have contributed to missed or delayed vaccination and widening immunity gaps during the study period. However, our study could not determine whether individual measles cases were directly related to vaccine stockouts or missed vaccination opportunities. This contrasts with findings from several low- and middle-income countries, where the pandemic caused sharp declines in routine immunization [46,47,48] due to health worker redeployment, supply chain disruptions, and reduced care-seeking behaviour. The resilience observed in our setting may reflect effective mitigation measures such as outreach campaigns and continued prioritisation of childhood immunization during the pandemic response.

The immediate 64% decline in confirmed measles cases at pandemic onset most likely reflects surveillance disruption rather than true reductions in transmission. Reduced health-seeking behaviour, redeployment of surveillance personnel, and diminished laboratory capacity likely drove underreporting, consistent with documented disruptions across sub-Saharan Africa [29]. The subsequent non-significant post-pandemic increase supports this interpretation, as cases rebounded sharply in 2022 and 2023 following surveillance recovery, likely representing a susceptible cohort that accumulated during the period of suppressed reporting and missed vaccinations. This pattern aligns with other interrupted time-series analyses across Ghana and the wider region. Ghana’s national tuberculosis and HIV surveillance data showed a comparable collapse at pandemic onset, with TB notifications falling 20.5% and HIV testing volumes falling 40.3% in April 2020, both attributed to reduced health-seeking behaviour rather than a true fall in disease burden [49]. A parallel analysis of Ghana’s essential health services found sustained declines in outpatient and antenatal care through late 2020, though first-dose pentavalent vaccination held steady, indicating that reporting disruption varied even within a single health system depending on service type [50]. Regionally, Togo recorded significant declines in six of nine HIV, malaria, and tuberculosis indicators immediately after pandemic onset [51], and Botswana saw sharp early-pandemic drops in HIV testing, diagnosis, and treatment initiation, with older age groups disproportionately affected, echoing the age-related pattern in the present study [52].

This signature was not universal, however. Malaria surveillance in Uganda showed no significant change in case counts or outpatient attendance after pandemic onset; disruption was confined to diagnostic testing and treatment prescription [53]. This divergence suggests that reporting disruption scaled with how central a disease’s surveillance was to routine facility operations, and how easily its diagnostic pathway survived restricted movement and reduced staffing. Measles surveillance, dependent on case-based investigation, specimen collection, and laboratory confirmation rather than passive facility attendance alone, was likely more exposed to the regional pattern of surveillance staff redeployment and reduced field investigation capacity than diseases detected through routine clinical presentation.

### 4.1. Strengths and Limitations

This study has several strengths. We used a nationwide case-based surveillance data and applied a robust interrupted time series framework with ARIMA error correction to account for autocorrelation in monthly data. Model selection relied on AIC, BIC, and residual diagnostics, which helped ensure well specified models. The analysis used 72 monthly observations over six years, which supports reliable trend estimation. It also combined descriptive analysis, spatiotemporal mapping, logistic regression, and ITSA. This multi-method approach offers a comprehensive view of measles epidemiology in Ghana and supports both clinical and program decisions.

However, some limitations remain. Some MR1 and MR2 coverage values exceeded 100%, suggesting inaccuracies in population estimates that may have introduced noise and masked true effects. The pre-pandemic period included only 26 months, which may have limited the precision of the estimated trends. ITSA also assumes that no other concurrent factors influenced the outcomes, although policy changes or outbreaks may have affected measles vaccination and surveillance during the study period. The ITS dataset comprised monthly aggregated data but did not include aggregated demographic characteristics. Therefore, we could not assess whether the magnitude or timing of pandemic-related changes differed across demographic groups. Similarly, urban or rural residence was not captured in the national surveillance database, limiting assessment of differences in healthcare access. Finally, the non-significant vaccination findings should be interpreted cautiously, given the wide confidence intervals.

### 4.2. Implications for Policy and Practice

These findings have several important implications for Ghana’s measles control programme. The sustained incidence above the WHO elimination target signals that current surveillance sensitivity is inadequate to detect and interrupt transmission chains before they spread across regions. Ghana’s surveillance system should be strengthened to meet the WHO benchmark. Additionally, the immediate 64% decline in confirmed measles cases during the active phase of the COVID-19 pandemic which likely reflects disruptions in case detection and surveillance, underscores the need for public health authorities to maintain essential surveillance functions for measles and other priority diseases during major outbreak responses to ensure timely case detection, sustain reporting, and prevent undetected transmission.

Nonetheless, the pre-existing and significant increasing trend in measles cases before the pandemic, combined with persistently suboptimal MR2 coverage, highlights that Ghana’s measles challenge is structural and not solely pandemic-related. Strengthening routine immunization performance in persistently low-coverage regions, implementing targeted catch-up immunization for missed children, and enhancing surveillance efficiency are all critical priorities. The directional decline in MR2 coverage at pandemic onset highlights the vulnerability of second-dose vaccines during health system disruptions and calls for specific protective strategies for later-schedule vaccines during future public health emergencies. Building resilient health systems capable of maintaining essential immunization services and surveillance during crises remains a critical priority for achieving Ghana’s 2030 measles elimination goal.

## 5. Conclusions

Ghana experienced high measles incidence driven by suboptimal routine immunization and COVID-19-related disruptions. Age, vaccination status, and region of residence were key predictors of infection. Vaccination among confirmed cases was suboptimal. National MR 1 and MR2 coverages were below the WHO elimination target of ≥95% with spatiotemporal variations, highlighting the need for targeted, region-specific interventions. To support progress toward the 2030 measles elimination goal, the Ministry of Health, through the Ghana Health Service, could strengthen routine immunization performance in persistently low-coverage districts, implement targeted catch-up immunization activities for missed children, and enhance surveillance efficiency, particularly case-based reporting completeness, timely case investigation, laboratory confirmation, and use of data for rapid response, to sustain population immunity and improve outbreak detection.

## Figures and Tables

**Table 1:** Demographic characteristics of confirmed cases of measles in Ghana, 2018–2023.

Variable	Frequency [N = 1688]	Percentage (%)
Median age (IQR) in years	2 (0–7)	
Age group		
<1	436	25.8
1–4	673	39.9
5–14	420	24.9
15–44	141	8.4
45+	18	1.0
Sex		
Female	800	47.4
Male	888	52.6
Outcome		
Alive	1686	99.8
Dead	2	0.1
Reporting Region		
Ahafo	72	4.3
Ashanti	104	6.2
Bono	38	2.3
Bono East	106	6.3
Central	27	1.6
Eastern	56	3.3
Greater Accra	59	3.5
Northeast	122	7.2
Northern	456	27.0
Oti	13	0.8
Savannah	96	5.7
Upper East	237	14.0
Upper West	84	5.0
Volta	53	3.1
Western	100	5.9
Western North	65	3.9
Year		
2018	34	2.0
2019	81	4.8
2020	88	5.2
2021	54	3.2
2022	460	27.3
2023	971	57.5

**Table 2:** Predictors of measles infection, Ghana, 2018–2023.

Variable	No measles n (%) 13,900 (89.2%)	Measles n (%) 1688 (10.8%)	cOR (95% CI)	aOR (95% CI)
Age group				
<1	2548 (85.4)	436 (14.6)	1.0	1.0
1–4	7691 (92.0)	673 (8.0)	0.51 (0.45, 0.58) ***	0.94 (0.81, 1.10)
5–14	2903 (87.4)	420 (12.6)	0.85 (0.73, 0.98) *	1.41 (1.19, 1.68) ***
15–44	643 (82.0)	141 (18.0)	1.28 (1.04, 1.58) *	1.74 (1.37, 2.22) ***
45+	115 (86.5)	18 (13.5)	0.91 (0.55, 1.52)	1.63 (0.93, 2.85)
Sex				
Female	6227 (88.6)	800 (11.4)	1.0	
Male	7673 (89.6)	888 (10.4)	0.90 (0.81, 1.00) *	0.98 (0.88, 1.10)
Vaccination status				
Not vaccinated	4672 (33.6)	1021 (60.5)	1.0	1.0
One dose	3845 (27.7)	203 (12.0)	0.24 (0.21, 0.28) ***	0.41 (0.34, 0.48) ***
Two doses	5383 (38.7)	464 (27.5)	0.39 (0.35, 0.44) ***	0.38 (0.33, 0.44) ***
Reporting Regions				
Ahafo	1184 (94.3)	72 (5.7)	1.0	1.0
Ashanti	1399 (93.1)	104 (6.9)	1.22 (0.90, 1.67)	1.06 (0.77, 1.47)
Bono	740 (95.1)	38 (4.9)	0.84 (0.56, 1.26)	0.77 (0.50, 1.17)
Bono East	584 (84.6)	106 (15.4)	2.98 (2.18, 4.09) ***	1.89 (1.35, 2.66) ***
Central	632 (95.9)	27 (4.1)	0.70 (0.45, 1.10)	0.86 (0.53, 1.38)
Eastern	1688 (96.8)	56 (3.2)	0.55 (0.38, 0.78) **	0.58 (0.40, 0.84) **
Greater Accra	1410 (96.0)	59 (4.0)	0.69 (0.48, 0.98) *	0.55 (0.38, 0.79) **
Northeast	155 (56.0)	122 (44.0)	12.94 (9.25, 18.11) ***	5.37 (3.73, 7.71) ***
Northern	788 (63.3)	456 (36.7)	9.52 (7.31, 12.40) ***	5.10 (3.84, 6.76) ***
Oti	227 (94.6)	13 (5.4)	0.94 (0.51, 1.73)	0.80 (0.43, 1.51)
Savannah	181 (65.3)	96 (34.7)	8.72 (6.19, 12.29) ***	4.41 (3.05, 6.39) ***
Upper East	683 (74.2)	237 (25.8)	5.71 (4.31, 7.55) ***	3.49 (2.58, 4.73) ***
Upper West	1252 (93.7)	84 (6.3)	1.10 (0.80, 1.53)	1.09 (0.77, 1.55)
Volta	1197 (95.8)	53 (4.2)	0.73 (0.51, 1.05)	0.55 (0.38, 0.81) **
Western	1353 (93.1)	100 (6.9)	1.22 (0.89, 1.66)	1.09 (0.79, 1.52)
Western North	427 (86.8)	65 (13.2)	2.50 (1.76, 3.56) ***	1.20 (0.83, 1.74)
Year				
2018	946 (96.5)	34 (3.5)	1.0	1.0
2019	2510 (96.9)	81 (3.1)	0.90 (0.60, 1.35)	0.84 (0.55, 1.27)
2020	1881 (95.5)	88 (4.5)	1.30 (0.87, 1.95)	1.08 (0.71, 1.64)
2021	2404 (97.8)	54 (2.2)	0.62 (0.40, 0.97) *	0.55 (0.35, 0.86) *
2022	3351 (87.9)	460 (12.1)	3.82 (2.68, 5.45) ***	2.72 (1.87, 3.95) ***
2023	2808 (74.3)	971 (25.7)	9.62 (6.78, 13.65) ***	6.29 (4.35, 9.09) ***

cOR = crude Odds Ratio, aOR = adjusted Odds Ratio, * *p* < 0.05, ** *p* < 0.01, *** *p* < 0.001.

**Table 3:** Predictors of vaccination status of confirmed measles cases, Ghana, 2018–2023.

Variable	Not Vaccinated n (%) 1021 (60.5%)	Vaccinated ^$^ n (%) 667 (39.5%)	cOR (95% CI)	aOR (95% CI)
Age group (in years)				
<1	328 (75.2)	108 (24.8)	1.0	1.0
1–4	368 (54.7)	305 (45.3)	2.52 (1.93, 3.28) ***	3.16 (2.37, 4.22) ***
5–14	219 (52.1)	201 (47.9)	2.79 (2.09, 3.72) ***	3.08 (2.25, 4.23) ***
15–44	96 (68.1)	45 (31.9)	1.42 (0.94, 2.16)	1.26 (0.80, 1.97)
45+	10 (64.4)	8 (36.1)	1.69 (0.55, 5.14)	1.47 (0.45, 4.78)
Sex				
Female	489 (61.1)	311 (38.9)	1.0	
Male	532 (60.0)	356 (40.0)	1.06 (0.87, 1.28)	
Regions				
Ahafo	36 (50.0)	36 (50.0)	1.0	1.0
Ashanti	49 (47.1)	55 (52.9)	1.12 (0.62, 2.05)	1.16 (0.60, 2.24)
Bono	22 (57.9)	16 (42.1)	0.73 (0.33, 1.61)	0.96 (0.40, 2.31)
Bono East	47 (44.3)	59 (55.7)	1.26 (0.69, 2.29)	1.29 (0.65, 2.56)
Central	13 (48.1)	14 (51.9)	1.08 (0.44, 2.61)	2.28 (0.87, 5.99)
Eastern	23 (41.1)	33 (58.9)	1.43 (0.71, 2.90)	2.15 (0.99, 4.65)
Greater Accra	39 (66.1)	20 (33.9)	0.51 (0.25, 1.04)	0.52 (0.24, 1.12)
Northeast	95 (77.9)	27 (22.1)	0.28 (0.15, 0.53) ***	0.36 (0.18, 0.73) **
Northern	299 (65.6)	157 (34.4)	0.53 (0.32, 0.87) *	0.53 (0.29, 0.94) *
Oti	10 (76.9)	3 (23.1)	0.30 (0.08, 1.18)	0.65 (0.15, 2.81)
Savannah	79 (82.3)	17 (17.7)	0.22 (0.11, 0.43) ***	0.39 (0.18, 0.86) *
Upper East	148 (62.4)	89 (37.6)	0.60 (0.35, 1.02)	0.42 (0.23, 0.78) **
Upper West	35 (41.7)	49 (58.3)	1.40 (0.74, 2.64)	2.08 (1.02, 4.22)
Volta	41 (77.4)	12 (22.6)	0.29 (0.13, 0.65) **	0.45 (0.19, 1.07)
Western	50 (50.0)	50 (50.0)	1.00 (0.55, 1.83)	1.20 (0.61, 2.35)
Western North	35 (53.8)	30 (46.2)	0.86 (0.44, 1.68)	0.68 (0.32, 1.44)
Year				
2018	21 (61.8)	13 (38.2)	1.0	1.0
2019	37 (45.7)	44 (54.3)	1.92 (0.85, 4.35)	1.60 (0.67, 3.83)
2020	43 (48.9)	45 (51.1)	1.69 (0.75, 3.79)	1.30 (0.53, 3.21)
2021	29 (53.7)	25 (46.3)	1.39 (0.58, 3.34)	1.22 (0.47, 3.17)
2022	368 (80.0)	92 (20.0)	0.40 (0.19, 0.84) *	0.42 (0.18, 0.94) *
2023	523 (53.9)	448 (46.1)	1.38 (0.69, 2.80)	1.96 (0.89, 4.34)

^$^ Table 3 identifies predictors of vaccination history among infected individuals (breakthrough infection), not determinants of vaccination coverage in the general population; cOR = crude Odds Ratio; aOR = adjusted Odds Ratio; * *p* < 0.05; ** *p* < 0.01; *** *p* < 0.001.

**Table 4:** Interrupted Time Series Analysis of the Impact of COVID-19 on MR1 and MR2 Vaccination Coverage, 2018–2023.

Parameters	MR1 Coefficient (95% CI)	*p*-Value	MR2 Coefficient (95% CI)	*p*-Value
Pre-intervention trend: Monthly change in coverage before COVID-19 (β_1_)	−0.21 (−1.44 to 1.02)	0.740	−0.10 (−1.20 to 1.00)	0.857
Intervention effect: Immediate change in coverage at COVID-19 onset (β_2_)	+1.13 (−19. to 21.51)	0.914	−3.66 (−27.04 to 19.71)	0.759
Post-intervention trend: Monthly change in coverage after COVID-19 (β_3_)	+0.23 (−1.13 to 1.58)	0.742	+0.26 (−1.03 to 1.55)	0.694
AR (1)	+0.32 (0.15 to 0.49)	<0.001	+0.39 (0.21 to 0.57)	<0.001
Seasonal AR (12)	Not applicable	—	+0.38 (0.02 to 0.74)	0.036
Residual SD	9.05	—	9.14	—
Final Model	ARIMA (1, 0, 0)	—	ARIMA (1, 0, 0) + SARIMA (1, 0, 0, 12)	—
AIC	533.6	—	538.9	—
BIC	547.3	—	554.9	—

MR1 model: ARIMA (1, 0, 0). MR2 model: ARIMA (1, 0, 0) with seasonal autoregressive term at lag 12, SARIMA (1, 0, 0, 12). Coefficients represent absolute percentage point changes in monthly vaccination coverage. AR (1) confirms significant first-order autocorrelation in both outcomes. Seasonal AR (12) for MR2 reflects significant annual cyclical patterns in second-dose coverage. CI: confidence interval; SD: standard deviation; AIC: Akaike Information Criterion; BIC: Bayesian Information Criterion.

**Table 5:** Interrupted Time Series Analysis of the Impact of COVID-19 on number of confirmed measles cases, 2018–2023.

Parameters	IRR	95%CI	*p*-Value
Pre-intervention trend: Slope of confirmed case before the COVID-19 (β_1_)	1.06	1.01–1.10	0.015
Intervention effect: Immediate change in confirmed case after the COVID-19 (β_2_)	0.36	0.17–0.75	0.007
Post-intervention trend: Change in slope of confirmed case after the COVID-19 (β_3_)	1.04	0.98–1.09	0.152
Sine1	1.27	0.95–1.71	0.105
Cosine1	0.94	0.65–1.35	0.742
Sine2	1.02	0.77–1.36	0.887
Cosine2	1.12	0.82–1.53	0.462

IRR: incidence rate ration; CI: confidence interval.

## Data Availability

The raw data supporting the conclusions of this article will be made available by the authors on request.

## Abbreviations

US CDC	Centers for Disease Control and Prevention
DALYs	Disability Adjusted Life Years
DSD	Disease Surveillance Department
EPI	Expanded Program on Immunization
GH-ERC	Ghana Health Service Ethics Review Committee
IDSR	Integrated Disease Surveillance and Response
IP	Immunization Programmes
MCV	Measles-Containing Vaccine
MMR	Measles Mumps and Rubella
MR	Measles-Rubella
QGIS	Quantum Geographic Information System
SORMAS	Surveillance Outbreak Response Management and Analysis System
USAID	United States Agency for International Development
WHO	World Health Organization
